# A Total Pleural Covering for Lymphangioleiomyomatosis Prevents Pneumothorax Recurrence

**DOI:** 10.1371/journal.pone.0163637

**Published:** 2016-09-22

**Authors:** Masatoshi Kurihara, Teruaki Mizobuchi, Hideyuki Kataoka, Teruhiko Sato, Toshio Kumasaka, Hiroki Ebana, Sumitaka Yamanaka, Reina Endo, Sumika Miyahashira, Noriko Shinya, Kuniaki Seyama

**Affiliations:** 1 Pneumothorax Research Center and Division of Thoracic Surgery, Nissan Tamagawa Hospital; 4-8-1 Seta; Setagaya-Ku; Tokyo, Japan; 2 The Study Group for Pneumothorax and Cystic Lung Diseases; 4-8-1 Seta; Setagaya-Ku; Tokyo, Japan; 3 Division of Respiratory Medicine, Juntendo University Faculty of Medicine and Graduate School of Medicine; 3-1-3 Hongo; Bunkyo-Ku; Tokyo, Japan; 4 Department of Pathology, Japanese Red Cross Medical Center; 4-1-22 Hiroo; Shibuya-Ku; Tokyo, Japan; 5 The Chemo-Sero-Therapeutic Research Institute (KAKETSUKEN); 1-6-1 Okubo, Kita-ku, Kumamoto-shi, Kumamoto, Japan; Peking University People's Hospital, CHINA

## Abstract

**Background:**

Spontaneous pneumothorax is a major and frequently recurrent complication of lymphangioleiomyomatosis (LAM). Despite the customary use of pleurodesis to manage pnenumothorax, the recurrence rate remains high, and accompanying pleural adhesions cause serious bleeding during subsequent lung transplantation. Therefore, we have developed a technique of total pleural covering (TPC) for LAM to wrap the entire visceral pleura with sheets of oxidized regenerated cellulose (ORC) mesh, thereby reinforcing the affected visceral pleura and preventing recurrence.

**Methods:**

Since January 2003, TPC has been applied during video-assisted thoracoscopic surgery for the treatment of LAM. The medical records of LAM patients who had TPC since that time and until August 2014 are reviewed.

**Results:**

TPC was performed in 43 LAM patients (54 hemithoraces), 11 of whom required TPC bilaterally. Pneumothorax recurred in 14 hemithoraces (25.9%) from 11 patients (25.6%) after TPC. Kaplan-Meier estimates of recurrence-free hemithorax were 80.8% at 2.5 years, 71.7% at 5 years, 71.7% at 7.5 years, and 61.4% at 9 years. The recurrence-free probability was significantly better when 10 or more sheets of ORC mesh were utilized for TPC (P = 0.0018). TPC significantly reduced the frequency of pneumothorax: 0.544 ± 0.606 episode/month (mean ± SD) before TPC vs. 0.008 ± 0.019 after TPC (P<0.0001). Grade IIIa postoperative complications were found in 13 TPC surgeries (24.1%).

**Conclusions:**

TPC successfully prevented the recurrence of pneumothorax in LAM, was minimally invasive and rarely caused restrictive ventilatory impairment.

## Introduction

Lymphangioleiomyomatosis (LAM), an uncommon neoplastic disease in women, is characterized by proliferation of atypical smooth muscle-like cells (LAM cells) and cystic destruction of the lungs [1, 2]. Pneumothorax is a common complication in LAM, since this disease involves the development of multiple fragile pulmonary cysts. In addition, pneumothorax often recurs as documented in the very high lifetime incidence of pneumothorax in LAM; for example, reports included 260 (66%) of 395 registered LAM patients from the LAM Foundation database in the USA [3] and 126 (73%) of 173 registered LAM patients from a nationwide, cross-sectional study in Japan [4]. Repeated pneumothoraces adversely affect quality of life for these patients and can cause pleural symphysis or chronic pneumothorax, often severely impairing lung function. Furthermore, pneumothorax can become life-threatening when accompanying an advanced stage of LAM. Therefore, effective treatment and management of pneumothorax as well as reduction of its recurrence are urgently needed.

Chemical and surgical pleurodesis has been utilized to prevent the recurrence of pneumothorax, but often fails in that approximately 30% recur [3]. Moreover, widespread and severe pleural adhesions sometimes result in restrictive ventilatory impairment, cause severe bleeding, and need long operation time when, as frequently follows, the patient undergoes lung transplantation [3, 5]. Almoosa et al. reported that 14 (18%) of 80 LAM patients who had lung transplantation experienced pleural-related postoperative bleedings, and 13 (93%) of them had prior pleurodesis [3]. Benden et al. additionally found that 29 (48%) of 61 transplant recipients had extensive pleural adhesions due to previous pleurodesis or pleurectomy; one-third of them had intra-operative moderate to severe hemorrhage and 4 had chylothoraces [5]. Clearly, an alternative to pleurodesis is desirable to prevent the recurrence of pneumothorax in patients with LAM, a representative disease among multiple cystic lung diseases that predispose their victims to repeated pneumothoraces.

Pneumothorax is a condition of air leakage into the pleural space from the diseased visceral pleura. In that context, we believe that the target of treatment should be confined to the visceral pleura without involving healthy parietal pleura. Therefore, we established a new surgical procedure, aptly named total pleural covering (TPC), by which the entire surface of the lung is covered with sheets of bioabsorbable material to reinforce the diseased visceral pleura without inducing severe pleural symphysis [6–8]. The purpose of this study is to analyze the results of TPC retrospectively in LAM patients as a strategy for the prevention of pneumothorax recurrence.

## Patients and Methods

### Animal experiments

To find a suitable material for reinforcing diseased visceral pleura without inducing adhesions between the visceral and parietal pleurae, the experimental study was conducted using Beagle dogs (6-month-old females, 8–10 kg). Three commercially available materials were tested: oxidized regenerated cellulose (ORC) mesh (Ethicon SURGICEL^®^ absorbable Hemostat gauze, Johnson & Johnson, Brunswick, NJ, USA), polyglactin 910 mesh (VIC) (Vicryl™, Johnson & Johnson, NJ, USA) and polyglycolic acid sheets (PGA) (NEOVEIL® sheet, Gunze, Tokyo, Japan). This animal experiment was carried out in strict accordance with the recommendations in the guideline for the care and use of laboratory animals and all efforts were made to minimize suffering. The protocol was approved by the CSTRI (the Committee of the Ethics for the Care and Use of Experimental Animals) of the Chemo-Sero-Therapeutic Research Institute (approval number B05-072).

After analgesic preparation with subcutaneous injections of carprofen (50 mg/ml, 0.088 mL/kg) and 0.05% atropine phosphate (0.5 mL), the animals were anesthetized with intramuscular injection of 20 mg of xylazine, followed by intramuscular injection of 30 mg of ketamine hydrochloride. Continuous intravenous injection of ketamine hydrochloride was also applied during the surgical procedure. Once deep anesthesia was achieved, hair of the chest and forelegs was shaved, and the body was fixed on the operation table in a left lateral decubitus position. After intratracheal intubation, 10 mg of suxamethonium chloride was administered with intravenous injection and mechanical ventilation (tidal volume, 200 ml; respiratory frequency, 14 breaths/min; and FIO_2_, 40%) was initiated. Open thoracotomy was performed with the incision from the right scapula to the second intercostal line, then the lung was pulled out from anterior to posterior lobes in series. Once the target lobe was fully inflated with an intratracheal pressure of 20 cmH_2_O, the lobe was kept inflated at constant pressure of 10 cmH_2_O. A surface part of the lung (2 x 0.5 cm in size and approximately 0.5 mm in depth) was excised with a scalpel to create an artificial air leakage as a model of pneumothorax. Once bleeding was controlled (an electrocautery was used if needed), the area of artificial air leakage was covered with a sheet of either ROC, VIC, or PGA followed by fibrin glue dripping [Bolheal^®^; Chemo-Sero-Therapeutic Research Institute (Kaketsuken), Kumamoto, Japan] according to the manufacturer's instruction (n = 3 for each group). After a chest drainage tube was placed, the lung was kept inflated with a constant intratracheal pressure of 20 cmH_2_O, and the chest was then closed with a skin stapler. Ten mg of ketoprofen was continuously administered for 24 hours. Once air leakage disappeared, the chest drainage tube was evacuated. Six weeks after surgery, the animals were sacrificed with overdosage of xylazine and ketamine, then autopsy was performed. Whenever pleural symphysis was observed, a part of the attached chest wall was resected en bloc with the lung. Specimens were fixed with 10% buffered formalin, and histological analysis was performed.

### Technical procedure for TPC in patients

**Fig 1:**
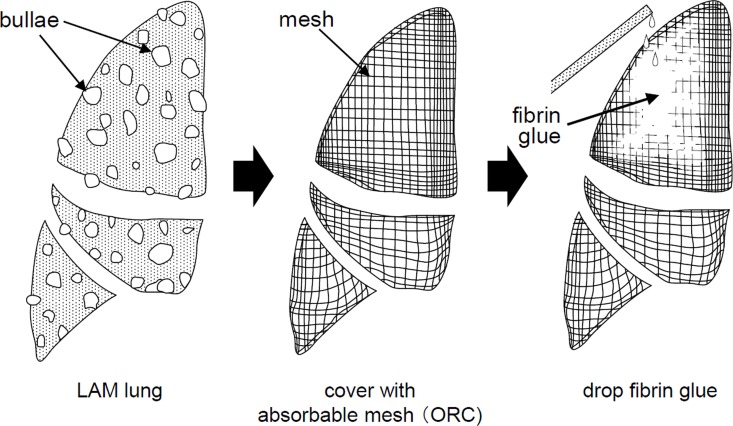
Schematic presentation of TPC workflow. TPC procedure for a LAM lung consists of 1) covering the lung’s entire visceral pleura with sheets of ORC mesh and 2) followed by spreading drops of fibrin glue. The entire procedure is performed under VATS.

Fig 1 depicts the workflow of TPC to totally enclose the entire lung surface with approximately 10 sheets of ORC mesh followed by fibrin glue dropping (Bolheal^®^).

With patients under general anesthesia and a separate ventilation system, TPC was performed by video-assisted thoracoscopic surgery (VATS) with a three- or four-port technique [12-mm port (Ethicon Endo-surgery; Endopath, Johnson & Johnson)]. A flexible LTF thoracoscope (Olympus Optical, Tokyo, Japan) was utilized throughout the entire surgical procedure. Since the LAM-afflicted lung is very fragile, the surgical intervention itself often causes *de novo* air leakage. Accordingly, the air leak test, performed by immersing the lung in saline, was applied with fastidious care.

**Fig 2:**
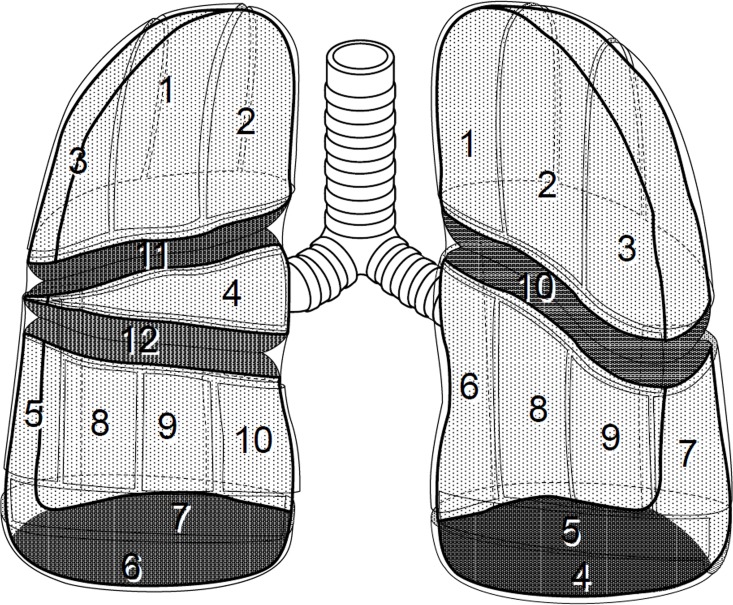
Numerical scheme for the order of covering lungs with ORC mesh under VATS. Each lung is depicted imaginarily as if frontal and lateral views were combined. Numbers indicate the standard order of placing sheets of ORC mesh over the entire visceral pleura in each lung. Beginning at the mediastinal surface of the left lung’s upper lobe, ORC mesh is placed as shown, subsequently laid at the basal area in the lower lobe and lastly positioned at interlobar and lateral surfaces. In the right lung, the surface of middle lobe is covered with ORC mesh just after the upper lobe’s surface is entirely covered.

Under halfway-inflation of the lung, the TPC was performed over the entire surface of the lung by delivering sheets of ORC mesh, forcing them into the thoracic cavity and affixing them onto the visceral pleura. Our TPC procedure started from an area where manipulation was technically difficult by VATS and then moved onto technically easier areas (Fig 2, S1 Video and S2 Video). Specifically, we first covered a mediastinal surface of the lung with ORC mesh, next moved onto the basal area in the lower lobe and lastly covered interlobar and lateral lung surfaces. Once the entire lung surface seemed to be covered by sheets of ORC mesh, the lung was fully inflated. This sequence always disclosed any uncovered lung surface area, and additional ORC mesh was used to ensure some overlap between each sheet of ORC mesh. Finally, fibrin glue (approximately 9 ml each of fibrinogen solution and thrombin solution) was applied drop by drop over the entire ORC surface. To complete the TPC procedure, a 20-Fr drainage tube was placed into the apex. Careful scrutiny confirmed that the edge bordering the lower lobe’s basal area was fully expanded. After the operation, the chest drainage tube was removed when the lung was fully expanded without air leakage and after drainage of pleural fluid decreased to less than 100 ml in a 24-hour period.

### Retrospective analyses of the LAM patients who had TPC

The medical records of LAM patients who underwent VATS for pneumothorax at Nissan Tamagawa Hospital between January 2003 and August 2014 were retrospectively analyzed. A total of 59 hemithoraces were from 47 operated LAM patients, 12 of whom had bilateral lung surgeries. TPC was indicated to avoid pleurodesis and/or repeated operations for LAM patients whose recurrent episodes of pneumothorax were not likely to be controlled with conventional treatment modalities. However, owing to severe pleural adhesions generated by prior chemical pleurodesis and/or surgeries in other hospitals, TPC was not completed in 5 hemithoraces from 4 LAM patients. Accordingly, the results of TPC applied for 54 hemithoraces from 43 LAM patients were analyzed in this study (Table 1). All these patients were Japanese women with histopathologically-confirmed LAM. The data collected were: ages when TPC was performed, subtype of LAM (TSC-associated or sporadic), number of pneumothorax recurrence(s) before and after TPC, and observation period. Postoperative complications were defined and graded according to the Clavien-Dindo classification of surgical complications [9]. The retrospective review of clinical data (all patient data were anonymous) was approved by the ethics committee of Nissan Tamagawa hospital (approval number TAMA2015007). All patients provided written informed consent. This retrospective study was conducted according to the principles expressed in the Declaration of Helsinki.

**Table 1:** Characteristics of study population (n = 43).

Male/Female		0/43
Age at TPC surgery	- median (range)	33 (21–53) ^a^
Clinical subtype of LAM	- n (%)	
TSC-associated LAM		8 (18.6)
Sporadic LAM		35 (81.4)
Use of oxygen supplementation	- n (%)	2 (4)

^a^ Since 11 patients underwent TPC bilaterally, a total of 54 surgeries were utilized for calculation.

### Statistical analysis

The probability of recurrence-free hemithorax after TPC is estimated by Kaplan-Meier analysis. TPC surgeries were categorized into two groups by the number of ORC sheets utilized: the group with 10 or more ORC sheets and the one with <10 sheets. The difference in the probability of no recurrence of pneumothorax between these two groups was examined by Cox regression model. Patient age and the categorized era when TPC was performed (2003–2007, 2008–2011, and 2012–2014) were included as covariates in the model. The frequency of pneumothorax (episodes/month) before and after TPC was analyzed using Wilcoxon signed rank test. Poisson regression analysis was also used to compare the frequency of pneumothorax before and after TPC with log [observation period (years)] as offset and subjects (intercept) as random effects. All tests were two-sided. All statistical analyses were performed using the statistical program R version 3.2.2. (http://cran.r-project.org). P<0.05 was considered statistically significant.

## Results

### Selection of a material suitable for pleural covering (animal experiment)

**Fig 3:**
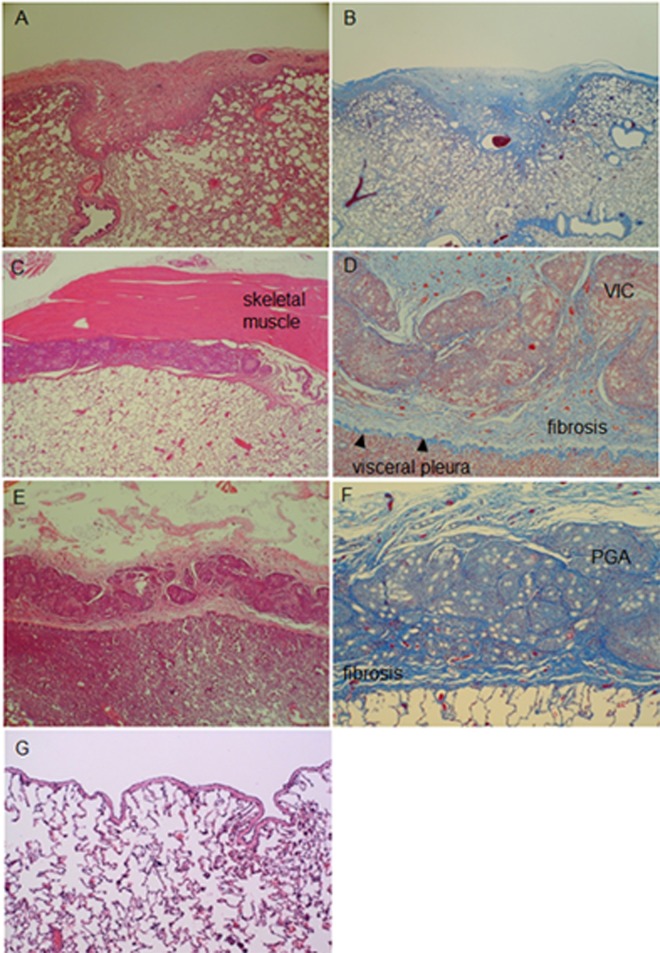
Selection of a material suitable for pleural covering. Microscopic findings of the visceral pleura in a Beagle dog 6 weeks after surgery to cover dog’s lung with either ORC mesh (A, B), VIC mesh (C, D) or PGA sheet (E, F), respectively. The photomicrograph of normal appearance of dog lung is presented (G) for comparison. The visceral pleura covered with ORC (A and B) was approximately 5 times thicker than untreated pleura. There was no adhesion between the visceral pleura and the chest wall. In contrast, VIC and PGA induced severe fibrotic changes associated with pleural symphysis (C, and E): Note that ORC was completely absorbed (B) whereas both VIC and PGA were not: the white dots indicate unabsorbable VIC mesh (D) or PGA sheet (F). A, C, E, andn G: Hematoxylin and eosin staining. B, D, and F: Masson-trichrome staining. A, B, C, E, andn G: low magnification view. D and F: high magnification view.

The aftereffects of pleural covering with ORC mesh, VIC mesh and PGA sheets followed by fibrin glue dripping were compared experimentally by using the canine model. Macroscopic observations revealed that both VIC mesh and PGA sheet induced severe pleural adhesion to the thoracic wall, whereas ORC mesh did not. Microscopic observation of the pleura 6 weeks after operation revealed that the visceral pleura covered with ORC mesh was approximately 5 times thicker than untreated portions, whereas extensive pleural symphysis accompanying fibroblast proliferation and collagen deposition was found after applying either VIC mesh or PGA sheets (Fig 3). Based on results from these animal experiments, we have determined to use ORC mesh for TPC in patients with LAM as well as for covering and reinforcing the resected areas of pleura when bullectomy was applied for primary spontaneous pneumothorax (PSP).

### Patients’ characteristics

Characteristics of the 43 LAM patients in this study are summarized in Table 1. All were Japanese women who had repeated pneumothoraces. Since 11 patients underwent TPC bilaterally, a total of 54 surgeries was performed. The left-to-right ratio of lung side requiring hemithorax operation was only slightly uneven: right side 32 (59%) and left lung 22 (41%). Operation times for the right and left lungs were 133.6 ± 34.9 and 127.4 ± 28.6 minutes (mean ± SD), respectively. The number of ORC mesh sheets (10.2 cm x 20.3 cm) utilized for TPC was 10.8 ± 2.5 sheets per patient (mean ± SD, right lung) and 9.7 ± 2.7 sheets per patient (left lung). All TPC procedures were performed as VATS without conversion into open thoracotomy.

### Pneumothorax recurrence per hemithorax after TPC

**Fig 4:**
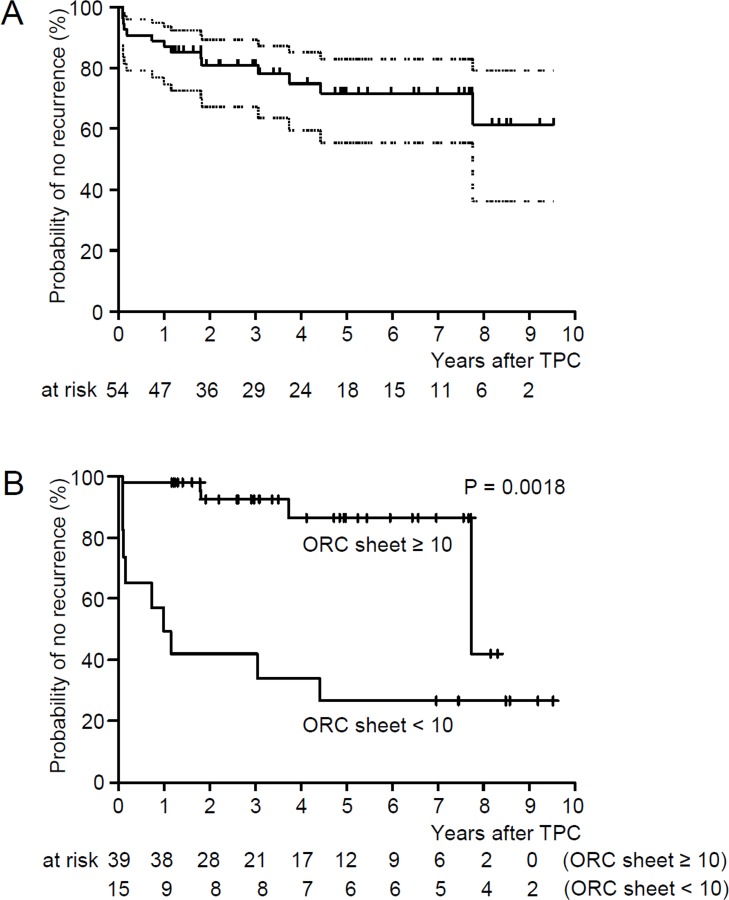
Kaplan-Meier estimate of the probability of recurrence-free hemithorax after TPC. A. All hemithoraces that had TPC (n = 54). The probability of recurrence-free hemithorax after TPC is 80.8% at 2.5 years, 71.7% at 5 years, 71.7% at 7.5 years, and 61.4% at 9 years. Dotted lines indicate the range of 95% confidence interval. B. Comparison of pneumothorax recurrence among hemithoraces where 10 or more ORC sheets were applied for TPC (n = 39) and those where fewer than 10 ORC sheets were used (n = 15) (P = 0.0018). The probability of no recurrence of pneumothorax after TPC was analyzed by Cox regression analysis adjusted for patient’s age and surgical skill by year (era) when TPC was implemented (3 categorized era: 2003–2007, 2008–2011, and 2012–2014).

After the TPC surgery, 14 hemithoraces from 11 patients underwent recurrences of pneumothorax. The time to the initial recurrence was 12.6 months (median, range 1.1–91.4 months). During the entire observation period after TPC (median 58.5 months, range 14.0–149.7), 40 of 54 hemithoraces (74.1%) remained free from recurrence. The probability of recurrence-free hemithorax after TPC, when estimated with the Kaplan-Meier method, was 80.8% at 2.5 years, 71.7% at 5 years, 71.7% at 7.5 years, and 61.4% at 9 years (Fig 4A). We expected that surgical success, i.e., no recurrence of pneumothorax after TPC, would be influenced mainly by the number of ORC sheets used for TPC and surgical skill at performing TPC. Since many subpleural cysts are at risk of rupture leading to pneumothorax in LAM, the number of ORC mesh sheets used for TPC is likely to influence the outcome. In our experience, at least 10 sheets of ORC mesh are needed to cover the entire visceral pleura in Japanese LAM patients, but pleural adhesions often hamper the placement of sufficient numbers of ORC sheets. On the other hand, the learning curve for applying the TPC technique provided an increasingly efficient surgical quality during the eleven years we have used this method compared to that in the early days. In this context, we compared the probability of recurrence-free hemithorax after TPC between the groups in terms of whether 10 or more ORC sheets were used for TPC (n = 39) or not (n = 15). Further, the eras when TPC was performed were adjusted; that is, they were arbitrarily categorized into the era of developing skill for TPC (2003–2007), that of maturing skill for TPC (2008–2012), and that of established skill for TPC (2012–2014). Finally, the age of each patient was factored into the outcomes. As shown in Fig 4B, the probability of no recurrence of pneumothorax after TPC was markedly better when 10 or more ORC sheets were used (P = 0.0018, Cox regression analysis).

### Comparison of pneumothorax episodes before and after TPC

**Fig 5:**
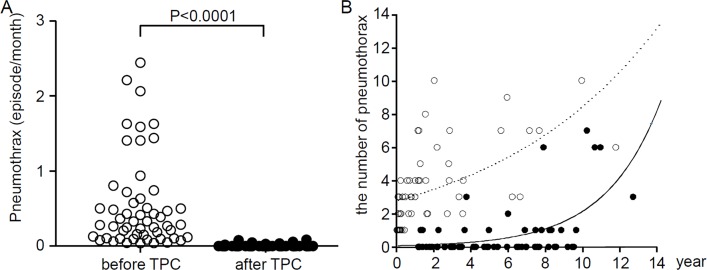
Comparison of the frequency of pneumothorax episodes before and after TPC. A. The number of pneumothorax episodes was divided by the observation period (the months from the first pneumothorax episode to TPC or those after TPC). The frequency of pneumothorax (episodes/month) was significantly reduced after TPC (P<0.0001). B. A Poisson regression model was used to compare the number of pneumothorax episodes before TPC (open circle) and after TPC (closed circle) with log [observation period (years)] as offset and subjects (intercept) as random effect (P<0.0001). The estimated regression equation of the number of pneumothorax before TPC is e^1.025 + 0.111×years^ before TPC (dotted line) and e^-2.606 + 0.337×years^ after TPC (solid line).

When the number of pneumothorax episodes and their frequency were compared before and after TPC, their incidence significantly decreased after TPC (Table 2 and Fig 5). We noted a total of 205 episodes of pneumothorax in 43 patients (in 54 hemithoraces) before TPC; 3.8 ± 2.6 episodes/patient (mean ± SD). The frequency of pneumothorax (episodes per month) before TPC was 0.544 ± 0.606 during the observation period of 13.92 months (median, range 0.48–138.94 months). In contrast, pneumothorax recurred in 14 hemithoraces from 11 patients as already mentioned; a total of 27 episodes of pneumothorax occurred after TPC. The frequency of pneumothorax after TPC was 0.008 ± 0.019 during the observation period of 58.47 months (median, range 13.97–149.71) (Table 2 and Fig 5A, P<0.0001). Furthermore, Poisson regression analysis demonstrated that the number of pneumothorax episodes after TPC decreased significantly as well (Fig 5B, P<0.0001).

**Table 2:** The number of pneumothorax episodes per hemithorax before and after TPC.

	Before TPC	After TPC	P
	(54 hemithoraces)	(54 hemithoraces)	
Pneumothorax (episode/month)	0.544 ± 0.606	0.008 ± 0.019	P<0.0001
	(0.038–2.447)	(0–0.087)	

Values are presented as mean ± SD (range).

The number of pneumothorax episodes during observation period (month) is determined per hemithorax before and after TPC. The observation period is 13.92 months (median, range 0.48–138.94 months) before TPC and 58.47 months (median, range13.97–149.71 months) after TPC.

ORC is widely recognized as an effective agent of postoperative adhesion-prevention. Its use has significantly reduced the incidence of postoperative adhesions after abdominal and gynecological surgeries [10–12]. Accordingly in our LAM patients who had recurrences of pneumothoraces after TPC, imaging studies revealed a partial pleural adhesion in the most of patients around the sites where ports were created. Although the majority of recurrent pneumothoraces were mild and spontaneously resolved under conservative observation, one patient required re-operation. Her thoracoscopy showed 1) whitish visceral pleura with diffuse thickening, and 2) focal pleural adhesions were visible around the apex, diaphragm, and the sites where ports were created at the preceding surgery, but they were easily dissociated (data not shown).

### Postoperative complications

Postoperative complications were found in 26 hemithoraces (48.1%); grade I, 1 hemithorax (1.9%); Grade II, 12 (22.2%); and Grade IIIa, 13 (24.1%). Grade IIIa complications consisted of 1) insertion of a chest tube for delayed occurrence of air-leakage (n = 3 hemithoraces at postoperative day (POD) 5, 11, and 15, respectively), 2) insertion of a chest tube for pleural effusion (n = 1), 3) looping of a persistent air-leakage site under local anesthesia (n = 1), 4) replacement of chest tube for prolonged air-leakage (n = 1), 5) wound infection (n = 1), and 6) thoracographic fibrin glue dropping [6] for delayed occurrence of air-leakage (n = 6).

### Lung function after TPC

We evaluated the effect of TPC on lung function (Table 3) in data from 38 patients (reviewed on average 11.8 months post-surgery). Of 10 patients who had bilateral TPCs, 5 patients were evaluated for post-TPC lung function after each TPC, and the remaining only after the second surgery. As an entire group, these patients experienced neither restrictive nor obstructive ventilatory impairment: the means of VC %predicted, and FEV_1_/FVC were 83.8% and 75.7%, respectively. In contrast, the mean of diffusing capacity was moderately impaired, presumably due to the underlying LAM. The results were similar, and no restrictive impairment was noted even when lung function data after the bilateral TPC were analyzed collectively (10 patients) (Table 3). Chronological changes in lung function before and after TPC surgery could not be determined, since no baseline data of lung function before TPC were available because of the patients’ repeated pneumothoraces.

**Table 3:** Results of lung function tests after TPC.

Months	VC %pred	FEV_1_/FVC	FEV_1_	FEV_1_%pred	DLCO/VA
after TPC	(%)	(%)	(L)	(%)	%pred (%)
All patients (38 patients, 43 hemithoraces)					
11.8 ± 5.9	83.8 ± 18.3	75.7 ± 17.8	2.01 ± 0.62	75.4 ± 23.1	63.7 ± 22.3
(3.2–27.5)	(46.1–127.9)	(32.2–95.4)	(0.42–3.11)	(16.7–111.2)	(25.8–106.3)
Patients with bilateral TPC (10 patients)					
10.7 ± 5.7	82.2 ± 15.2	70.0 ± 21.5	1.85 ± 0.58	68.2 ± 21.5	51.6 ± 21.1
(3.2–18.7)	(62.4–108.7)	(32.2–92.4)	(0.67–2.58)	(24.6–98.6)	(29.7–92.3)

Data are presented as means ± SD (range).

## Discussion

The results of our retrospective analysis presented here clearly demonstrate that TPC is an effective way of preventing the recurrence of pneumothorax without severe impairment of lung function. Recurrent pneumothorax is one of the major complications of LAM [1–4]. Owing to the morbidity and excessive costs associated with multiple recurrences in these patients, early intervention with pleurodesis has been recommended despite the increased risk of perioperative bleeding involved [3]. Additionally, severe pleural adhesions caused by chemical and/or mechanical pleurodesis administered to such patients who later undergo lung transplantation frequently increase the latter procedure’s technical difficulty and cause serious bleeding [3, 5, 13]. Furthermore, pleurodesis often causes restrictive impairment of lung function.

Lunge et al. reported that primary pneumothorax subjects treated with talc poudrage had greater impairment of lung function than those managed with simple drainage [14]. A still more serious concern associated with chemical or mechanical pleurodesis is its failure to attain pleural adhesion throughout the entire visceral surface of the lung; instead, the results can be a patchy, focal, and incomplete outcome of pleural adhesion. In the latter situation, a major problem remains for subsequent surgical treatment when pneumothorax recurs, which actually is a frequent sequel. Unlike primary spontaneous pneumothorax, LAM involves a far greater likelihood of recurrences because, in this neoplastic disease, the number of cysts gradually increases over time, resulting in destruction of lung parenchyma and severe respiratory failure [1, 2]. Therefore, thoracic surgeons have long awaited an alternative treatment that provides an improvement over pleurodesis in the prevention of recurrent pneumothorax for patients with LAM, whose victims are exceptionally prone to repeated incidences of pneumothorax.

As an innovative procedure, we utilized TPC to manage pneumothoraces in LAM patients for the purpose of preventing subsequent recurrences by thickening diseased visceral pleura with no pleural adhesions or minimal even if generated. In LAM, multiple fragile cysts appear diffusely on the surface visceral pleura, and each of these cysts is a potential source of initial or recurrent pneumothorax. Thus, a standard surgical approach such as the resection of the affected lung tissue does not control the virtually inevitable recurrences. We considered that TPC applied to reinforce the fragile visceral pleura with ORC mesh would be well-suited for both the management of pneumothorax and the prevention of recurrence.

A variety of covering materials such as polyglactin 910 mesh (Vicryl^TM^, Johnson & Johnson) and polyglycolic acid (PGA) sheets (NEOVEIL® sheet, Gunze, Tokyo, Japan) and ORC mesh have been applied for the reinforcement of visceral pleura to stop air leakage during lung surgeries for primary and secondary pneumothorax or lung volume reduction surgery for COPD [15–17]. However we have noted empirically through our own surgical experiences when treating primary spontaneous pneumothorax that the lung surface area covered with ORC mesh is reasonably well-thickened with minimal or no pleural adhesion (S1 Fig). Our clinical observation is also supported by the fact that ORC sheets have been utilized as adhesion barriers for abdominal and gynecological surgeries and proved to reduce the incidence of adhesions [10–12]. To the contrary, we frequently found severe adhesions during surgical treatment of patients with recurrent pneumothorax in whom PGA sheets were previously used as the covering material at other hospitals in Japan. Those experiences prompted us to conduct animal experiments (data not shown) to determine the best material for TPC from two viewpoints: 1) sufficiently reinforcing diseased visceral pleura and 2) inducing no pleural adhesions at all or at least very few. On the basis of these experiences and considerations, we selected ORC as a most suitable material for TPC to reinforce fragile visceral pleura. Nevertheless, PGA sheets may suffice as a covering material for topical use, since it seems be ideal for staple-line reinforcement and reliable in controlling air leakage [15]. Meanwhile, Noda et al. described a modified TPC for a LAM patient with intractable bilateral pneumothoraces [7]. By taking advantage of PGA sheets’ adhesion-inducing property, they first applied PGA sheets for staple-line reinforcement and then performed TPC with ORC mesh [7, 8]. However, we remain concerned about whether the combination of ORC plus PGA as covering materials is absolutely free from the potential for causing severe pleural adhesion, since no adequate data are currently available. Therefore, further investigation with a large number of patients is warranted.

The outcome of TPC is likely to depend whether the entire visceral surface of the lung is fully covered or not. This is supported by Cox regression analysis for the probability of recurrence-free hemithorax after TPC between groups in whom 10 or more sheets of ORC mesh were or were not applied. The probability outcome was adjusted by patient age and the era when TPC was performed as a representative of surgical skill. Depending on lung size though, 10 sheets of ORC mesh were generally sufficient to cover the entire lung surface in Japanese LAM patients. However, complete coverage over an entire lung surface was sometimes hampered by the presence of very firm pleural adhesions to which synechiotomy was unattainable. Here again, the important factor to consider for treating pneumothorax secondary to diffuse cystic lung disease like LAM is that diseased or fragile parts of visceral pleurae are apt to rupture diffusely and focally on the lung surface. Therefore, procedures inducing pleural symphysis should be avoided, because they seldom obtain pleural adhesion over the whole lung surface. Apparently, the reverse of important tip for successful outcome of TPC would be the technical limitation of TPC. The existence of a patchy, focal, and incomplete outcome of pleural adhesion that was generated naturally or by the preceded treatments like chemical pleurodesis, precludes successful application of TPC. With standard surgical skills for VATS and several experiences by practice, well-trained thoracic surgeons would perform TPC with an expecting outcome. This is supported by the fact that TPC is gradually popularized and its successful applications for intractable pneumothorax other than LAM have already been reported by other institutes in Japan [8, 18, 19].

In conclusion, we applied TPC for patients’ LAM-afflicted lungs by using ORC mesh to reinforce their fragile visceral pleura. As surgical outcomes, very small or no pleural adhesions developed. Our results advocate TPC as an effective method to prevent the recurrence of pneumothorax without causing a significant loss of lung function.

## Supporting Information

S1 Fig Thoracoscopic appearance of the visceral pleura covered with ORC mesh. A male 19-year-old patient had a spontaneous pneumothorax on the right side of the lung. Bullae at the apex of upper lobe and the superior segment of lower lobe were resected, and each staple line of resection was reinforced with sheets of ORC mesh. Thirteen months later, the right-sided pneumothorax recurred, and VATS was then performed as treatment. A. Thoracoscopic view at re-operation. No pleural adhesion was noted. The area of visceral pleura that had been covered 13 months previously looked white and inhomogeneously thickened. B. Histopathological examination revealed that the area of visceral pleura formerly covered with ORC mesh was thickened with fibrous tissues (hematoxylin-eosin stain).
(TIF)

S1 Video Supporitng video for TPC procedure: S1 Video shows TPC applied to the left pneumothorax in a patient with sporadic LAM (35-years-old female). The entire procedures were performed under video-assisted thoracoscopic surgery (VATS). We abridged the time greatly for short presentation. This representative procedure displays coverage of the left upper lobe’s surface. A thoracoscope turns around from the lower lobe to the upper lobe to enable examination of the entire surface of the left lung. The numerous cysts with pinkish color are easily recognized. Generally speaking, the LAM lungs are so fragile due to cystic destruction that they are easily damaged by being held with surgical forceps. Accordingly, we routinely handle them as gently as possible without actually holding them. We start TPC at the apical part of the left upper lobe by placing a sheet of ORC mesh (correspond to No. 1 of the left lung in Fig 2) and then move to the other areas in a standard order (see Fig 2). Each sheet of ORC mesh should be placed to partially overlap at the margin of adjoining sheet so that the entire lung surface is covered sufficiently with ORC meshes when the lung fully expands. Once the whole surface of left upper lobe is covered with ORC mesh, fibrin glue is applied drop by drop to spread over the covered lung surface.
(WMV)

S2 Video Supporitng video for TPC procedure: S2 Video shows TPC applied to the left pneumothorax in a patient with sporadic LAM (35-years-old female) (the same patient with Video 1). The entire procedures were performed under video-assisted thoracoscopic surgery (VATS). We abridged the time greatly for short presentation. This representative image shows a review of coverage and final adjustment at the end of the entire procedure. After the whole surface of left lower lobe is covered according to the standard order (see Fig 2), careful observation over the whole lung surface is needed to identify if any area becomes uncovered after lung inflation, especially on the basal part of lower lobe and interlobal surface. In this patient, the apical part (S6 area, corresponding to No. 7 of the left lung in Fig 2) of the left lower lobe was partially uncovered after lung expansion. Then an additional sheet of ORC mesh is placed over the opening (the view shows an apical part of lower lobe and thoracic wall). Turning the thoracoscope to look up from the caudad to the cephalad direction confirmed that the entire lung surface is fully covered with ORC meshes (a part of lower lobe and the whole upper lobe are represented). After the fibrin glue is dropped, a chest tube is placed to complete the operation.
(WMV)
