# Supplementary figures and images for: A Total Pleural Covering for Lymphangioleiomyomatosis Prevents Pneumothorax Recurrence
Source: PLoS One. 2016 Sep 22;11(9):e0163637. doi: 10.1371/journal.pone.0163637 (PMC5033523; doi:10.1371/journal.pone.0163637)

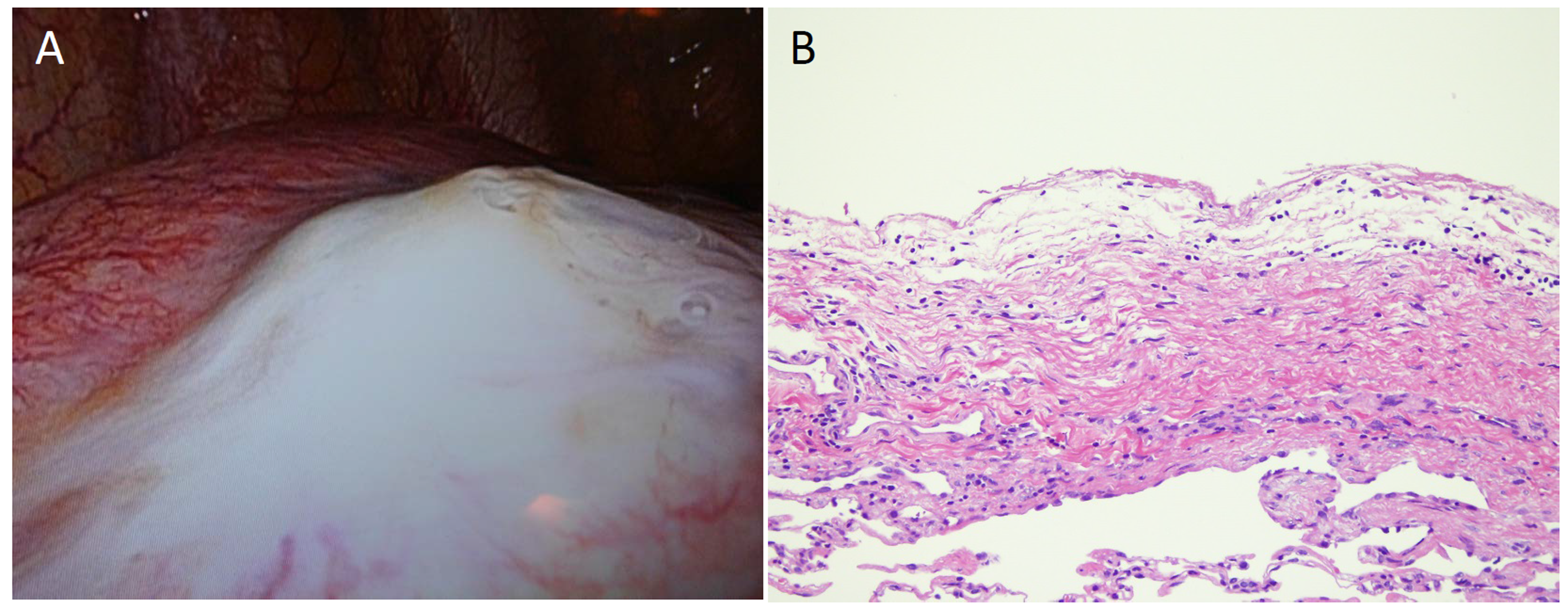

Supplement: S1 Fig — A male 19-year-old patient had a spontaneous pneumothorax on the right side of the lung. Bullae at the apex of upper lobe and the superior segment of lower lobe were resected, and each staple line of resection was reinforced with sheets of ORC mesh. Thirteen months later, the right-sided pneumothorax recurred, and VATS was then performed as treatment. A. Thoracoscopic view at re-operation. No pleural adhesion was noted. The area of visceral pleura that had been covered 13 months previously looked white and inhomogeneously thickened. B. Histopathological examination revealed that the area of visceral pleura formerly covered with ORC mesh was thickened with fibrous tissues (hematoxylin-eosin stain). (TIF) [file pone.0163637.s001.tif]
